# Breeding and Domesticating Crops Adapted to Drought and Salinity: A New Paradigm for Increasing Food Production

**DOI:** 10.3389/fpls.2015.00978

**Published:** 2015-11-12

**Authors:** Ana Fita, Adrián Rodríguez-Burruezo, Monica Boscaiu, Jaime Prohens, Oscar Vicente

**Affiliations:** ^1^Instituto de Conservación y Mejora de la Agrodiversidad Valenciana, Universitat Politècnica de ValènciaValencia, Spain; ^2^Mediterranean Agroforestal Institute, Universitat Politècnica de ValènciaValencia, Spain; ^3^Institute of Plant Molecular and Cellular Biology, Universitat Politècnica de València – Consejo Superior de Investigaciones CientíficasValencia, Spain

**Keywords:** food security, abiotic stress, breeding methods, salt tolerance, drought tolerance, biotech crops, biotechnology

## Abstract

World population is expected to reach 9.2 × 10^9^ people by 2050. Feeding them will require a boost in crop productivity using innovative approaches. Current agricultural production is very dependent on large amounts of inputs and water availability is a major limiting factor. In addition, the loss of genetic diversity and the threat of climate change make a change of paradigm in plant breeding and agricultural practices necessary. Average yields in all major crops are only a small fraction of record yields, and drought and soil salinity are the main factors responsible for yield reduction. Therefore there is the need to enhance crop productivity by improving crop adaptation. Here we review the present situation and propose the development of crops tolerant to drought and salt stress for addressing the challenge of dramatically increasing food production in the near future. The success in the development of crops adapted to drought and salt depends on the efficient and combined use of genetic engineering and traditional breeding tools. Moreover, we propose the domestication of new halophilic crops to create a ‘saline agriculture’ which will not compete in terms of resources with conventional agriculture.

## Introduction

**FIGURE 1:**
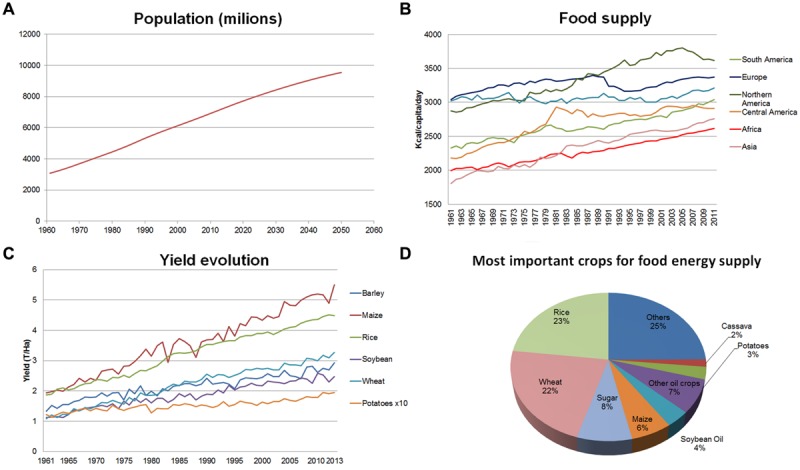
**Global population, food supply and crop yield trends. (A)** World population since 1960 to 2050. **(B)** Food supply (Kcal/capita/day) in different regions of the world since 1961 to date. **(C)** Yield evolution for primary crops since 1960. **(D)** Most important crops for food energy supply. All data taken from FAO-STAT (http://faostat3.fao.org/home/E). Figures ellaborated by the authors from FAOSTAT data (FAOSTAT, 2015).

Current world population is about 7.2 × 10^9^ people and is projected to grow by almost 30% over the next 35 years, to reach 9.2 × 10^9^ individuals by 2050 (**Figure 1A**). FAO estimates indicate that it will be necessary to increase agricultural production by at least 60% over 2005–2007 levels to meet the expected demand for food (Alexandratos and Bruinsma, 2012). Looking back at the recent past this goal, *a priori*, does not seem so difficult to be achieved. Indeed, in the last 50 years, specifically between 1960 and 2009, world population more than doubled while it was still possible to increase the amount of food per capita, from 2200 Kcal/person/day to an average of more than 2800 Kcal/person/day. This means that today enough food is produced to feed everyone living on this planet. Clearly, this food is not distributed evenly: while food supply in Europe reached in 2009, on average, almost 3400 Kcal/person/day, the mean figures for Africa were below the 2600 Kcal/person/day level (**Figure 1B**; FAOSTAT, 2015). A fairer distribution of food worldwide is largely a matter of political will and international solidarity. In consequence it is frequently argued that there will be no technical problems to feed the world’s population if we are able to share the available food resources. Unfortunately, this reasoning is flawed when taking a look on our present agricultural systems, the challenges posed by the climate change and the need of sustainability.

### Beyond the Green Revolution: Present Situation and Challenges for the Near Future

Between the 1940s and 1970s, scientific and technical advances induced a new trend in agricultural practices that enabled growers to increase crop yields dramatically. Such movement was later known as the ‘Green Revolution’ (GR; Borlaug and Dowswell, 2005). The first major innovation of the GR consisted in the development of new high-yielding, disease-resistant (mainly to stem rust), and semi-dwarf wheat cultivars (Dubin and Brennan, 2009) which then was spread to other crops. Another key point of the GR was the monoculture practice and the replacement of traditional agricultural methods by modern approaches and technologies, including the massive use of agrochemicals (pesticides, herbicides, chemical fertilizers), mechanization of labor, and a large increase in the area of irrigated crop land (Evans, 1998). This new way of conceiving agriculture boosted the food production of primary crops and has resulted in increasing yields every year (**Figure 1C**).

Despite the undeniable positive effects of the GR, there is also a negative side of the strategies used in the past, which may hamper further increases in food production under the present, quite different circumstances. In many cases, the agricultural systems established during the GR have evolved toward an excessive use of intensive production practices that may not be sustainable. They include, among others: greenhouses for continuous production of certain commodities all around the year, lack of crop rotation, the massive and uncontrolled use of synthetic fertilizers, or the cultivation in semi-arid regions of species with high water requirements, which need large amounts of irrigation water. These practices may cause a series of problems for agricultural production in the near future, such as the appearance of new pathogens and pests and also the depletion, contamination, and salinization of soils and ground waters (Shiva, 1991; Dehaan and Taylor, 2002; McDonald and Linde, 2002). In addition, the use of fertilizers from non-sustainable or non-renewable sources (e.g., synthetic fertilizers, mineral phosphate) may also limit crop yields when their availability decreases in the medium or long term (Shiva, 1991; Stewart et al., 2005).

In addition, GR caused direct impacts on diet and on diversity. On the one hand, staple crops were improved and the total amount of protein and energy available to people was increased, but not the nutritional value in terms of micronutrients; this, together with the reduction of the variety of products consumed (specially by poor people) led to an increase of the micronutrient malnutrition which is known as ‘hidden hunger’ (Welch and Graham, 2002). On the other hand modern uniform cultivars with high yields and resistances to many pests and diseases gradually substituted traditional cultivars, heirlooms, and local varieties. This situation was detrimental to the use and conservation of the latter, and many of those cultivars were lost forever (Hammer and Khoshbakht, 2005). That is, GR has favored the global predominance of a narrow range of crop species and cultivars (**Figure 1D**). Moreover, modern cultivars frequently encompass a low genetic diversity themselves as seed companies usually restrict their breeding materials to a very limited genetic pool. The process of gradual (and irreversible) decline of agricultural diversity, and consequently of their gene pool, i.e., ‘genetic erosion,’ may jeopardize food production in the future (FAO, 2010). Thus, the loss of genetic diversity decreases the opportunities to find new sources of variation to fight future challenges (e.g., new pests and diseases or new races in already known pests and diseases, changing environments) to which modern varieties will not provide resistances or tolerances (Esquinas-Alcázar, 2005).

Apart from the aforementioned reasons concerning crop varieties and agricultural techniques, many other circumstances will hamper the necessary increase in food production in the years ahead (Vermeulen et al., 2012). First, the forecasted effects of global climate change, with an increase in mean temperatures worldwide and more frequent, longer and more intense extreme weather phenomena, such as droughts, ‘heat waves,’ or floods, will obviously affect negatively overall agricultural production (IPCC, 2014). The temperature increase may allow cultivation of some crops in regions which were previously too cold to grow them, and a higher atmospheric CO_2_ concentration will stimulate photosynthesis and plant growth, but these side effects will not compensate at all the general reduction of crop yields worldwide (Bita and Gerats, 2013). Lack of rain and higher temperatures are already contributing to the spreading of desertification, mostly in arid and semi-arid regions, and the situation is expected to worsen in the near future. Similarly, an increasing problem also exacerbated by climate change is the loss of irrigated cropland due to ‘secondary’ soil salinization (see below) (Connor et al., 2012). There is also an increasing demand for biofuels, which are at present obtained from food crops and produced in arable land and thus competing with food production (Searchinger et al., 2015). Therefore, it is imperative to develop and implement new strategies for improving agricultural production worldwide.

### Strategies to Increase Global Food Production

#### Increasing the Global Cropland Area

One of the simplest ways to improve agricultural production with the current technology and crop cultivars would be to significantly extend the global cropland area. However, new agricultural lands are scarcely available or can only be obtained at a high environmental cost. Also, land cultivated with irrigation is much more productive than rainfed cropland. Irrigation systems are currently used to grow crops in about 280 × 10^6^ ha. of arable land (Fischer et al., 2008); this represents just under 20% of the total cultivated land, but produces more than 40% of world food supplies (Munns and Tester, 2008). Therefore, a significant increase in the area of irrigated arable land would lead to a parallel increase in food production. Unfortunately, this will not be possible in a world where fresh water for irrigation is becoming an increasingly scarce resource. Another possibility would be the use of natural habitats of great ecological value, such as rainforests, but it would be against the necessary sustainability of natural resources and conservation of biodiversity. The use of our actual high-input agriculture in marginal, low-fertility land will be also unsustainable, as it will require large inputs of agrochemicals and could not be maintained for a long time. In addition, at the moment the area available for agriculture is actually being reduced, mostly by the change of land use due to urban development and industrialization in many emerging economies and developing countries.

#### Increasing Area of Biotech Crops

Once the possibilities of significantly increasing the overall area of cultivated land or the relative area of irrigated land are ruled out, there is still room for improving crop yields by extending the arable land used to grow biotech crops, since they have higher average productivity than their conventional counterparts as shown by the assessment of the global economic impact of GM crops from the beginning of their large-scale commercial cultivation in 1996 (Brookes and Barfoot, 2014; Klümper and Qaim, 2014) and despite some studies indicate the contrary in specific cases (Elmore et al., 2001; Ma and Subedi, 2005). In 2014, the total area of GM crops was 181.5 million hectares, which represents about 13% of global farmland (James, 2014). Yet the rate of adoption of the main current GM food crops (herbicide tolerant, and/or insect resistant soybean, maize and rapeseed) is already very high in the major producing countries, leaving little, or no room for expansion. Therefore, an important increase in the general productivity of these crops could only occur in those countries with low adoption rate (James, 2014). Another way to augment total agricultural production could be based on increasing the cultivation of GM varieties of ‘minor’ crops that at present occupy a very small proportion (<1%) of global biotech arable land. Nevertheless, only about half of the extra profit obtained by farmers growing GM crops is due to their higher productivity, and the rest because of savings in labor and energy costs (Brookes and Barfoot, 2014). Therefore, the expected increase in the worldwide cultivation area of our present biotech crops may contribute, but probably only to a limited extent, to the needed increase in food production in the next decades. In addition, genetic transformation is carried out on previously improved, ‘GR-derived’ crop varieties, so that cultivation of those GM plants does not solve the aforementioned drawbacks and limitations of our present agricultural systems, regarding lack of biodiversity, high inputs requirements, or nutritional and sustainability issues.

A step forward, and another possibility to improve agricultural production, is the cultivation of ‘second generation’ biotech crops where the introduced traits are related to nutritional aspects rather than yield. Among other examples, we can mention the iconic ‘golden rice’ which synthesize β-carotene in the endosperm (Ye et al., 2000). Also the development of a GM maize expressing the enzyme phytase in the seeds, which are used as feed for monogastric animals (pigs, poultry); this modification allows increasing the assimilation of inorganic phosphate making growth and meat production more efficient, while decreasing the environmental pollution caused by the animals manure (Chen et al., 2008).

#### Increasing Yield under Abiotic Stress Condition

For all crops, average yields are only a fraction of maximum potential yields or even of record yields actually obtained under optimal cultivation conditions. These differences are mostly due to abiotic stresses affecting the plants growing in the fields, such as water or salt stress, wind, hail, flooding, cold, high temperatures, ozone, UV irradiation, etc. All these stressful conditions cause yield losses which can range from about 50%, for sugar beet or potato, to more than 80%, as it is the case for sorghum or wheat (Buchanan et al., 2000). Drought and high soil salinity, in particular, are the major causes reducing crop productivity, and hence food production worldwide (Bartels and Sunkar, 2005).

Drought is the single stressful environmental factor most devastating for agriculture. Inadequate rainfall brings about a progressive decrease in the amount of available water in the soil, affecting plant growth and development and reducing crop yields; prolonged periods of drought cause premature plant death and the complete loss of the crop and, eventually, the abandonment of the land. This problem will worsen in the near future in arid and semiarid regions, the most affected by the forecasted effects of climate change, which include the occurrence of longer, more frequent and more intense drought periods (IPCC, 2014). In fact, almost 50% of the earth’s land surface is arid or semiarid, but cropland in these regions is actually the most productive, as far as enough water is available for irrigation. If drought means that irrigation is needed to allow efficient crop growth, prolonged irrigation brings another serious problem for agriculture: soil salinization. At present, more than 20% (and up to 50% according to some estimates) of irrigated cropland is affected by salt, to a greater or lesser extent (Owens, 2001; Flowers, 2004). After years or decades of continuous irrigation, toxic ions dissolved in irrigation water (even if fresh, good-quality water is used) progressively accumulate in the soil, leading to this ‘secondary salinization’ – of anthropic origin– which causes the loss of more of 10 million hectares of arable land every year (Owens, 2001); these losses are expected to increase in the years ahead, again because of the foreseeable effects of climate change. In addition to the loss of agricultural land due to secondary salinization, there are large areas of naturally saline and alkaline soils, amounting to about 6% of the world’s land surface. These marginal lands have never been cultivated because of their high soil salinity, as our present major crops are all salt-sensitive.

If varieties of our major crops are bred for abiotic stress tolerance (AST), especially with enhanced tolerance to drought and high soil salinity, it is evident that there is a wide margin for the improvement of crop productivity. Availability of drought tolerant crops will allow growing them in arid and semiarid lands, providing reasonable yields without depending on irrigation water, or at least with reduced irrigation. These varieties could even help to recover agricultural abandoned farmland where cultivation of conventional crops is not profitable due to the low yields obtained. Similarly, salt tolerant crops will have a positive effect on crop productivity in irrigated agriculture; despite progressive soil salinization, these plants could maintain stable yields, and could also be grown using brackish water for irrigation – thus saving good-quality fresh water for human consumption and other uses. Salt-tolerant crops will also help to reclaim former arable land already lost due to secondary salinization (Owens, 2001), or could even be grown in naturally saline, marginal soils.

Summarizing, drought and salt-tolerant crops may significantly contribute to increase crop productivity and food supply by reducing yield losses and extending the area available for agriculture. In order to develop these tolerant varieties in the shortest possible time, all possible strategies should be used: genetic engineering and generation of transgenic plants, conventional breeding with the help of the new biotechnological tools available to the breeder and domestication and/or use of halophytes. These three issues are going to be discussed in the following sections.

## Drought and Salt Tolerant GM Crops

### Searching for ‘Stress Tolerance’ Genes

**FIGURE 2:**
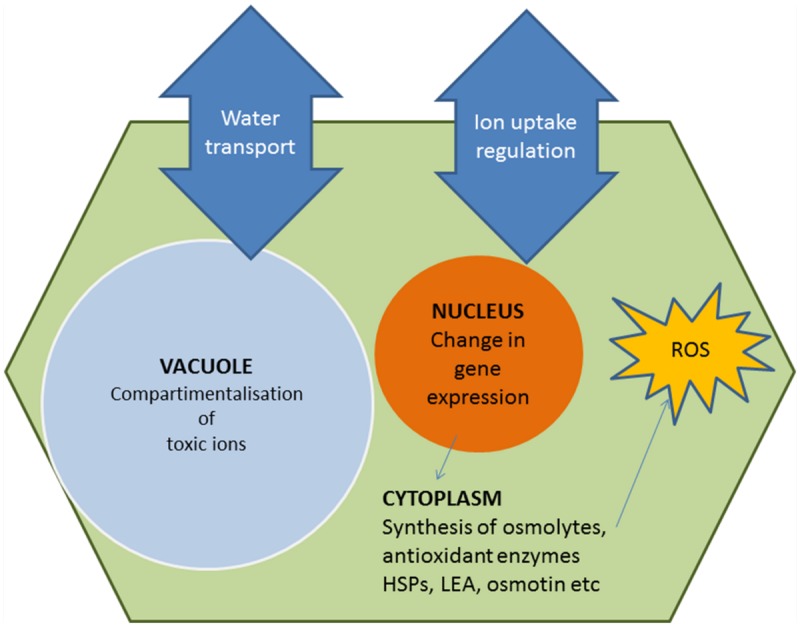
**Diagram of basic and general stress responses caused by high soil salinity or drought.** Under stress, plant cells try to counteract cellular dehydration through ion homeostasis and osmotic balance (Zhu, 2001; Munns and Tester, 2008; Kronzucker and Britto, 2011). Osmolytes not only contribute to osmotic adjustment, but also play ‘osmoprotectant’ roles (Ashraf and Foolad, 2007; Chen and Murata, 2008; Flowers and Colmer, 2008; Szabados and Savouré, 2010). They act as low-molecular-weight chaperons, directly stabilizing macromolecular structures and have additional functions in the mechanisms of response to stress, as ‘reactive oxygen species’ (ROS) scavengers, signal transduction elements or molecules for the storage of C and/or N and energy to be used during recovery from stress. The diagram has been elaborated by the authors.

A lot of effort has been invested in the last 20–30 years in the isolation and characterization of genes involved in stress response pathways (**Figure 2**), with the expectation that their overexpression in transgenic plants would confer improved tolerance to abiotic stress. They include, for example, genes encoding ion transporters or enzymes of osmolyte biosynthesis. Moreover, most stressful environmental factors induce an increase in cellular ‘reactive oxygen species’ (ROS) levels thus causing, as a secondary effect, oxidative stress in plants. Consequently, another basic and general response to abiotic stress involves the activation of enzymatic and non-enzymatic antioxidant systems to mitigate oxidative damage of DNA, membranes and proteins. All these responses are mediated by major stress-induced changes in gene expression patterns, which also include the synthesis of specific ‘protective’ proteins, such as heat-shock proteins (HSPs), late embryogenesis-abundant (LEA) proteins, osmotin, etc. There are 100s of reports in the literature describing how the expression of these genes may confer variable levels of tolerance to drought, salinity, high temperatures, and/or other abiotic stresses to the GM plants. Many of these papers are mentioned in a number of reviews published over the last years (e.g., Apel and Hirt, 2004; Wang et al., 2004; Battaglia et al., 2008; Chen and Murata, 2008; Miller et al., 2008; Ashraf, 2009; Türkan and Demiral, 2009; Szabados and Savouré, 2010; Gil et al., 2013); a few specific examples are briefly described below, and some more are summarized in **Table 1**.

**Table 1:** Some examples of improved stress resistance through genetic transformation.

Function/transgene		Source organism	Host species	Phenotype	Reference
Ion transporters	*AlNHX*	*Aeluropus littoralis*	*Nicotiana tabacum*	ST^∗^	Zhang et al., 2008a
	*SbSOS1*	*Salicornia brachiata*	*N. tabacum*	ST	Yadav et al., 2012
	*GmHKT*	*Glycine max*	*N. tabacum*	ST	Chen et al., 2014
Osmolyte synthesis	*Bv*CMO	*Beta vulgaris*	*N. tabacum*	DT	Zhang et al., 2008b
	*SeCMO*	*Salicornia europea*	*N. tabacum*	ST	Wu et al., 2010
	*betA*	*Escherichia coli*	*Zea mays*	DT	Quan et al., 2004
	*AtP5CS*	*Arabidopsis thaliana*	*Petunia hybrida*	DT	Yamada et al., 2005
	*OsP5CS*	*Oryza sativa*	*P. hybrida*	DT	Yamada et al., 2005
	*P5CS*	*Vigna aconitifolia*	*Triticum aestivum*	DT	Vendruscolo et al., 2007
	*mtlD*	*E. coli*	*T. aestivum*	DT, ST	Abebe et al., 2003
	*PsTP*	*Pleurotus sajor-caju*	*N. tabacum*	DT	Han et al., 2005
	*otsA* and *otsB*	*E. coli*	*O. sativa*	DT, ST, LTT	Garg et al., 2002
Transcription factors	*DREB1A/CBF3*	*A. thaliana*	*Festuca arundinacea*	DT	Zhao et al., 2007
	*AtDREB1A*	*A. thaliana*	*Arachis hypogaea*	DT	Bhatnagar-Mathur et al., 2007
	*MYB15*	*A. thaliana*	*A. thaliana*	DT	Ding et al., 2009
	*AP37*	*O. sativa*	*O. sativa*	DT	Oh et al., 2009
Protective proteins (LEA, HSP)	*sHSP17.7*	*O. sativa*	*O. sativa*	DT	Sato and Yokoya, 2008
	*HVA1*	*Hordeum vulgare*	*Morus indica*	DT, ST	Lal et al., 2008
	*BhLEA1* and *2*	*Boea hygrometrica*	*N. tabacum*	DT	Liu et al., 2009
Antioxidant activity	*VTE1*	*A. thaliana*	*N. tabacum*	DT	Liu et al., 2008
	*APX5*	*A. thaliana*	*N. tabacum*	DT, ST	Badawi et al., 2004
	*MnSOD*	*Pisum sativum*	*O. sativa*	DT	Wang et al., 2005

*^∗^ST, salt tolerant; DT, drought tolerant; LTT, low temperature tolerant.*

#### Ion Transporters

One of the first reports on improvement of salt tolerance in transgenic plants overexpressing ion transporters refers to *Arabidopsis thaliana* transformed with the *AtNHX1* gen from the same species, encoding a vacuolar Na^+^/K^+^ antiporter (Apse et al., 1999). Later-on, promising results were obtained when the same gene was expressed in tomato plants, which appeared to become highly tolerant to NaCl and accumulated salt in the leaves, but not in the fruits; that is, the quality of the harvested product was not affected (Zhang and Blumwald, 2001); however, these results were soon challenged since they could not be reproduced by other authors (Cuartero et al., 2006). Overexpression of a different Na^+^/K^+^ antiporter, this time of the plasma membrane (*AtSOS1*) also increased salt tolerance in transgenic *Arabidopsis plants* (Shi et al., 2003). More recent research has demonstrated, again in *A. thaliana*, that the tissue-specific strong expression of the sodium transporter gene *AtHKT1;1* can reduce shoot Na^+^ accumulation and therefore also improve salt tolerance (Møller et al., 2009).

#### Osmolyte Accumulation

Another approach to generate stress-resistant GM plants was based on the manipulation of specific metabolic pathways, by overexpression of the appropriate enzymes, to increase the cellular levels of particular osmolytes. For example, expression of Δ1-pyrroline-5-carboxilase synthetase in transgenic tobacco led to increased (10- to 18-fold) levels of proline and a significant improvement of drought and salt tolerance, as compared to the non-transformed controls (Kishor et al., 1995). Similarly, transgenic rice transformed with the *codA* gene for choline oxidase showed increased levels of glycine betaine and a parallel enhancement of tolerance to cold and salt stress (Sakamoto et al., 1998). There are also several reports describing the generation of transgenic plants with improved tolerance to drought, cold, and/or salt stress, correlated with an increase in the intracellular contents of the disaccharide trehalose, for example in tobacco (Romero et al., 1997), potato (Yeo et al., 2000), or rice (Garg et al., 2002). One of the first reports supporting a functional role of osmolytes in salt stress tolerance mechanisms described the transformation of tobacco with the *mt1D* gene, isolated from *Escherichia coli* and encoding the enzyme mannitol-1-phosphate dehydrogenase; its expression in the GM tobacco led to increased mannitol contents and improved salt tolerance, as compared with the control plants (Tarczynski et al., 1992, 1993). Similar results were obtained by Zhifang and Loescher (2003), who transferred to *A. thaliana* the mannose-6-phosphate reductase gene from celery, as an alternative to the bacterial gene.

#### Overexpression of Antioxidant Enzymes and Other Proteins

Activation of antioxidant systems is one of the general responses of plants to different abiotic stresses, and there are several reports on transgenic plants with enhanced stress tolerance by expression of different antioxidant enzymes, such as glutathione *S*-transferase/glutathione peroxidase in tobacco (Roxas et al., 1997), or superoxide dismutase in alfalfa (McKersie et al., 1999).

Other proteins whose expression in transgenic plants confers tolerance to different abiotic stresses include, to give only a few examples: heat shock proteins (HSPs) such as *Arabidopsis* At-HSP17.6A, which enhances osmotolerance when overexpressed in the same species (Sun et al., 2001); LEA proteins, such as HVA7 from barley, which confers water and salt stress tolerance in transgenic rice (Xu et al., 1996); or transcription factors which control the expression of other genes involved in abiotic stress responses, for example expression of DREB1A in *Arabidopsi*s (under the control of a stress-inducible promoter) improved plant drought, salt and freezing tolerance (Kasuga et al., 1999).

A slightly different strategy to enhance stress tolerance in GM plants relies on the overexpression of stress ‘target’ proteins; that is, proteins which are inactivated under stress conditions or which are functionally relevant in cellular processes inhibited by stress. For example, more than 10 years ago two genes encoding SR-like splicing factors were isolated from an *A. thaliana* cDNA library, based on the salt tolerance conferred when expressed in yeast; their expression in transgenic *Arabidopsis* plants led to salt (LiCl and NaCl) tolerance (Forment et al., 2002). A few years later, it was shown that these GM plants were also markedly resistant to drought (Bourgon et al., 2007). These results suggested that RNA processing – or RNA metabolism, in general – is very sensitive to abiotic stress, and provided new possible targets for engineering tolerance in plants.

Despite these and many other promising results, the usefulness of the aforementioned genes as biotechnological tools for developing stress-tolerant biotech crops has been questioned (Flowers, 2004), for several reasons: (i) assessment of tolerance phenotypes in *in vitro* systems, which do not reflect the natural physiological conditions of the plants; (ii) lack of quantitative data regarding the differences in tolerance between the transgenic and the control plants; (iii) evaluation of the plants only at specific phases of development, not along their full life cycle; (iv) side effects of expression of the transgenes, under non-stress conditions, causing reduced growth or developmental abnormalities, etc. Nevertheless, the most serious criticism is that in most of these experiments model species, such as *A. thaliana* or *Nicotiana tabacum* have been used, and generally it is not possible to extend the results to crop species. In any case, stress tolerance has been seldom evaluated from an agronomic point of view, and it is important to note that any improvement in tolerance is useless if the quality of the harvested product (seeds, fruits, tubers...) or the crop’s yield is significantly reduced.

Referring specifically to the generation of salt-tolerant crops, intensive research is being carried out in many public and private labs all over the world and it is to be expected that this goal will be achieved in the medium or long term using genetic engineering techniques. Yet at present no commercial salt-tolerant ‘biotech’ (GM) crops are growing in our fields.

### Drought Tolerant Maize, A Successful Case

Contrary to salt-tolerant biotech crops, which are not yet available, the first drought tolerant GM crop has been commercially launched not long ago, in 2012. Monsanto, in collaboration with BASF, has developed a genetically modified maize variety with improved resistance to water stress, conferred by expression of bacterial genes encoding RNA chaperones (Castiglioni et al., 2008); this reinforces the idea that maintaining active RNA metabolism is critical for plant performance under stress. After going through all the regulatory process and field trials, the company obtained approval in USA and Canada, and the crop was grown for the first time in 2012, in the more drought-prone U.S. states of Nebraska and Kansas. The expected increments in yield under normal conditions were very modest, of no more than 10%. However, that year there was a very strong drought which devastated the crops of conventional maize in non-irrigated farmland, while the biotech variety performed quite well. Nevertheless, some further improvement is expected with more advanced ‘versions’ of the crop and by introducing the trait in other, more drought-tolerant cultivars obtained by classical breeding. There are plans to make drought tolerant maize available to some sub-Saharan African countries by 2017, in the frame of a Public–Private Partnership project entitled ‘Water Efficient Maize for Africa (WEMA)’, coordinated by the African Agricultural Technology Foundation, based on Nairobi (James, 2014). Development of this drought-resistant maize has open the way to introduce the trait in other major crops, although overexpression of some of the genes mentioned in the previous section may also be successful in delivering crops with enhanced resistance to water deficit.

In any case, it is to be expected that biotech crops tolerant to different environmental stresses will be available in the near future for large-scale commercial cultivation. Those with improved tolerance to drought and high soil salinity, the abiotic stress conditions responsible for most of the reduction in crop yields worldwide, will significantly contribute to the much needed increase in food production in the next decades.

## Conventional Breeding for AST

### Present Situation of AST Breeding

Conventional breeding for adaptation to abiotic stresses is far more complicated than breeding for other traits. One reason is the difficulty to establish the characters which best define tolerant genotypes. For each stress there are different levels and mechanisms of tolerance, which can also produce divergent responses depending on the plant phenological stage (Reynolds et al., 2005). In addition, these traits are controlled by numerous genes that generate a continuous variation, the so-called ‘quantitative trait loci’ (QTL; Collins et al., 2008). Despite these limitations and drawbacks, conventional breeding for AST has proven successful in some cases in the past. For example, Ashraf (2010) listed a series of new cultivars tolerant to drought, bred by controlled mating and selection. Traditional breeding was also successful to breed new salt-tolerant rice varieties (Singh et al., 2010).

Success in classical breeding relays on the one hand in a proper identification of a donor of tolerance genes. Landraces and neglected crops exhibit a great genetic diversity and different survival strategies, displaying a large variation in their responses to stress (Reynolds et al., 2005, 2007). They have been selected for centuries by the farmers to be adapted to a particular environment. Therefore, landraces which evolved along different conditions differ in their adaptation in those conditions. For example, geographical gradients of rainfall should produce a gradient of drought resistance in the germplasm, as shown by Blum and Sullivan (1986), who were able to identify superior genotypes for drought performance in sorghum and millet which came from the driest regions within their study area. In a 2-year experiment with wheat cultivars and landraces from different countries, Dencic et al. (2000) identified genotypes with high yields under optimal cultivation conditions and under drought stress. Therefore, given their potential to contribute with favorable alleles to stress tolerance, landraces have been included along with other cultivars in many screenings for AST. Interesting sources of resistance against a broad range of abiotic stresses have been found in different species; for example, for drought tolerance in beans (Muñoz-Perea et al., 2006), chickpea (Anbessa and Bejiga, 2002), wheat (Mobasser et al., 2014), maize (Cairns et al., 2013), oat (Sánchez-Martín et al., 2012), or potato (Barra et al., 2013); for salt tolerance in tomato (Foolad, 2004) or rice (Yeo et al., 1990).

The second basis for a successful breeding for AST is the identification of QTL responsible for the tolerance and their association to linked molecular markers, which are then used for an effective selection through marker assisted selection (MAS). In the last decades, numerous studies have been conducted to map genes or QTL associated, for example, to drought tolerance in rice (Courtois et al., 2000; Price et al., 2002; Bernier et al., 2007), barley (Talamé et al., 2004), or maize (Tuberosa et al., 2002), or to salt resistance in rice (Lin et al., 2004) or soybean (Li et al., 2005). Yet the transference of marker-QTL information to breeding programs is still limited mainly due to: (i) the dependence of the QTL effects on genetic background and the environment, which implies that it is necessary to repeat the experiment in different conditions/years and different genetic backgrounds to obtain reliable results, and (ii) the possible linkage drag of undesirable traits when wild relatives are used as QTL donors (Podlich et al., 2004; Vargas et al., 2006). The new genomic approaches have overcome many of the restrictions for the detection and characterization of QTL/genes responsible for AST (Kole et al., 2015). Next generation sequencing (NGS) technologies allow the massive discovery of molecular markers to obtain ultra-high density genetic maps, which are very useful to locate precisely the QTL and clone them. In addition, using markers close to the QTL minimizes the linkage drag during the introgression process. Moreover, SSR and especially SNPs generated through NGS can be used in high-throughput genotyping platforms, which permit the simultaneous analysis of many markers and many individuals. Therefore it is possible to move from the exploitation of recent recombination through the analysis biparental mapping populations to the genome-wide association (GWA) studies, which use the natural diversity to identify genetic loci associated with phenotypic trait variation and provides better resolution. For example, this strategy resulted in the identification in barley of a number of genomic regions that strongly influenced salt tolerance and ion homeostasis (Long et al., 2013). Genomic studies in combination with transcriptomic analysis also allow discovering new genes and regulatory systems and their positions (Roorkiwal et al., 2014). All this information, available from large public databases, grants the transfer of information from one species to another.

These molecular technologies offer a wide array of tools to accelerate AST breeding. However, phenotyping protocols suitable for the evaluation of large populations are essential, both for the identification of sources of tolerance and in the selection process. Nowadays there are many new phenotyping tools, based on non-invasive or minimally invasive techniques, which can be implemented in phenotyping platforms able to acquire large amounts of data (Fiorani and Schurr, 2013). Nevertheless, experiments under controlled conditions (greenhouse), require careful planning (e.g., pot size, growth medium, water and nutrient supply, light quantity, etc.) to ensure both within-laboratory repeatability and reliability with respect to field results (Poorter et al., 2012a,b). On the field the challenge is the optimal platform to ensure high quality records. Field phenotyping tools based on remote sensing of growth-related parameters, using spectral reflectance and infrared thermometry to estimate plant water status are available (Masuka et al., 2012). Sophisticated biometrical methods are necessary to calibrate and validate the data and build robust prediction models (Cabrera-Bosquet et al., 2012; Römer et al., 2012). In practical breeding programs, however, time for data validation for selection can be extremely short, e.g., only one to two months between harvest and sowing.

### The Way to Follow: Some Examples of Successful Breeding for AST

The combination of appropriate selection of sources of resistance, MAS, precise phenotyping protocols, and other new molecular tools open a promising scenario for the improvement of plant AST. In the following paragraphs, a few examples of the successful development of tolerant cultivars are described.

At CIMMYT, Mexico, a marker-assisted backcross (MABC) selection program, meant to improve grain yield under limited water conditions, was carried out in tropical maize, and involved crossing a drought resistant line with a drought susceptible line. After the backcross program, under severe drought conditions five MABC-derived hybrids produced yields about 50% higher than those of control hybrids (Ribaut and Ragot, 2006).

Azucena, an upland japonica rice variety originally from the Philippines identified as drought tolerant (Courtois et al., 1996) was used as donor parent to improve root morphological characteristics of Kalinga III, an Indian upland indica elite rice variety that escapes end-of-season drought through early maturity; yet it is still susceptible to early and mid-season drought (Steele et al., 2006). Again a MABC breeding program was used to pyramid four previously reported QTLs for improved root morphological characteristics (Price et al., 2000, 2002) from Azucena into Kalinga III. The resulting NILs were evaluated in field trials and four of them resulted superior in terms of tolerance (Steele et al., 2007). The result of this breeding program was a highly drought tolerant rice variety, Birsa Vikas Dhan 111 (PY 84), released in the Indian state of Jharkhand (Steele, 2009).

‘Saltol’ is a major QTL for salt tolerance in rice, which maps in the short arm of chromosome 1 (Thomson et al., 2010). MAB strategy was undertaken to introgress the ‘Saltol’ QTL into the widely accepted two mega rice varieties BR11 (T. Aman, monsoon) and BR28 (Boro, dry, winter). For ‘Saltol’ QTL introgression, a near isogenic line (NIL) derived from Pokkali (a salt tolerant donor variety) was used (Linh et al., 2012).

In the case of the development of salt tolerant durum wheat, Byrt et al. (2007) and Munns et al. (2012) explored natural diversity in shoot Na^+^ exclusion within ancestral wheat germplasm. Durum wheat (*Triticum turgidum* ssp. *durum*) is more salt sensitive than common bread wheat (*Triticum aestivum*). Both species are polyploids, durum wheat a tetraploid (genomes A and B), and bread wheat a hexaploid (genomes A, B, and D). In the D genome there is a Na^+^ excluding locus (*Kna1*) enabling bread wheat to maintain relatively low leaf Na^+^ concentration, which contributes to salt tolerance. A source of Na^+^ exclusion (the sodium transporter *Nax2*) not present in durum wheat or in bread wheat was found in the wheat relative *T. monococcum*. This latter species contains an A^m^ genome, which is homologous to the A genomes (although some recombination/pairing problems exist) of *T. turgidum* ssp. *durum* and *T. aestivum* but, as it has evolved separately, contains many genes not present in durum or bread wheat. *Nax2* was introgressed from *T. monococcum* into a modern durum cultivar, Tamaroi, by means of durum derivative line 149 and NILs with and without *Nax2*. The newly developed durum wheat lines with the introgression of *Nax2* were tested in saline fields, giving yields approximately 25% higher than the controls.

These are just a few examples of the possibilities that appear when a good characterization of the germplasm meets with a breeding-oriented use of modern molecular tools. These results encourage the use of conventional breeding to generate AST varieties of our crops, which will contribute – together with genetically modified abiotic stress tolerant plants – to the future food supply needs.

## Halophytic Crops For A ‘Saline Agriculture’

**FIGURE 3:**
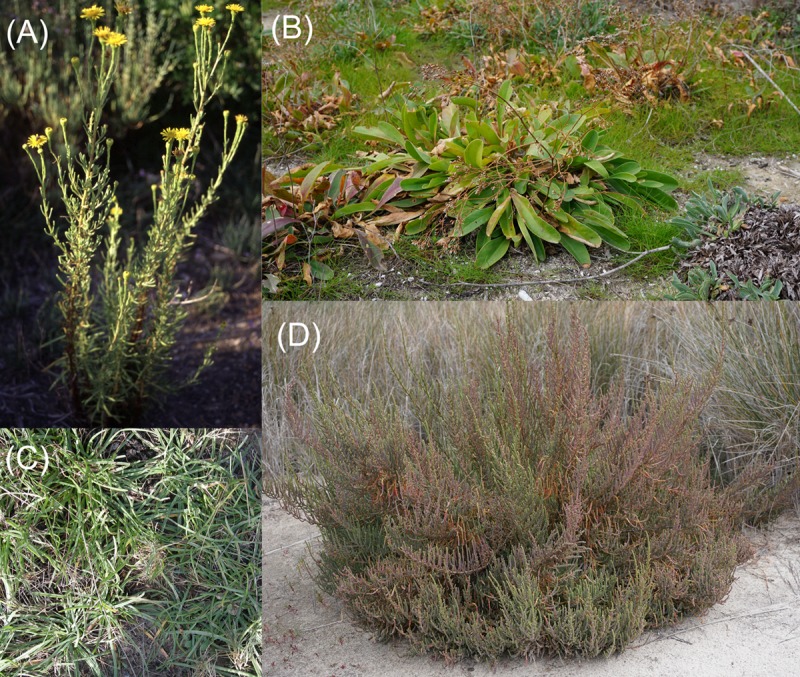
**Image of some plants suitable for a saline agriculture. (A)** *Inula crithmoides*, **(B)** *Limonium girardianum*, **(C)** *Plantago crassifolia*, and **(D)** *Sarcocornia fruticosa*. Pictures belong to the authors.

Halophytes are defined as plants specific of saline environments that are able to survive and complete their life cycles in the presence of salt concentrations equivalent to, at least, 200 mM NaCl (Flowers et al., 1986; Flowers and Colmer, 2008). However, many can grow at salt concentrations even higher than that of seawater. Only about 0.25% of all angiosperm species are considered to be halophytic, which still represents more than 600 taxa, widely distributed among different plant genera and families (Flowers et al., 2010). Many of these halophytes (**Figure 3**) have the potential to be transformed into useful ‘new’ crops although, as wild plants, they should go first through a domestication process. Nevertheless, as they are already salt tolerant – which is the most important and the most difficult trait to introduce and manipulate – it should be relatively easy to carry out specific breeding programs to rapidly improve the required agronomic characteristics of the most promising halophytic taxa. It may be necessary to select the best genotypes for particular cultivation conditions, to increase yields, to eliminate or at least reduce the content of toxic compounds or anti-nutrients (e.g., saponins, Glenn et al., 1991), or to improve marketing characteristics (shelf-life, market availability, uniformity of the product in size, color, taste, etc.), and to tailor general agricultural methods to specific crops. In short, some of the common objectives of traditional plant breeding and agricultural practice.

For centuries, many halophytes have been collected by people from nature, to be self-consumed, grown in backyard and kitchen gardens or sold in local markets; their leaves are commonly eaten as raw vegetables (in fresh salads, for example) or, in some cases, also cooked or pickled. The traditional use as food of these species will make them more easily acceptable by the general public, so that they are appropriate candidates to be domesticated and transformed into leafy vegetable crops for saline agriculture. These plants are not only edible but, in general, also very nutritious: they are rich in protein, antioxidants and essential nutrients – minerals, vitamins, amino acids, and/or fatty acids (**Table 2**).

**Table 2:** Some examples of halophilic species and their main uses.

Main use	Species	Features	Reference
Vegetable	*Inula crithmoides*	Source of iodine in the diet	Zurayk and Baalbaki, 1996; Tardío et al., 2006
	*Aster tripolium*	High levels of polyphenols and minerals	Koyro et al., 2011
	*Atriplex hortensis*	High protein and amino acid contents	Carlson and Clarke, 1983
	*Plantago coronopus*	Vitamins A, C and K	Koyro, 2006
	*Batis maritima*	Essential amino acids and antioxidants such as vitamin E	Debez et al., 2010
	*Portulaca oleracea*	High levels of omega-3 fatty acids and several antioxidant compounds (β-carotene, vitamins C and E)	Simopoulos, 2004
Vegetable, grain crop and oilseed	*Salicornia and Sarcocornia* sp.	Rich in essential fatty acids, minerals, and antioxidant compounds such as polyphenols	Ventura and Sagi, 2013
Grain crop	*Distichlis palmeri Chenopodium quinoa*	High-quality protein	Glenn et al., 2013
Oilseed	*Suaeda fruticosa, Haloxylon stocksii, Halopyrum mucronatum, Cressa cretica, Arthrocnemum macrostachyum, Alhaji maurorum*	22–25% of oil content and relatively high fraction of unsaturated fatty acids	Weber et al., 2007
Feed fodder livestock	*Atriplex lentiformis*		Glenn et al., 2013

Regarding the halophilic plants that have a greater potential to be domesticated and cultivated commercially as vegetables, we should mention species of the genera *Salicornia* (annuals) and *Sarcocornia* (perennial), which are closely related taxonomically. These taxa have attracted attention for its long tradition of use as food by people, and its extreme salt tolerance, as they can grow in the presence of seawater. Several field trials have been conducted in different countries to optimize their culture conditions, with promising results, and several projects are in progress for the small-scale, commercial cultivation of this species using seawater for irrigation, for example in Mexico, run by private companies or sponsored by non-profit organizations, such as the OASE Foundation^1^ or the Seawater Foundation.^2^ Given this accumulated knowledge and experience, it should be relatively simple to develop breeding programs for transforming *Salicornia* into a ‘standard’ crop, based on the selection of the best genotypes and the improvement of its marketing characteristics (Ventura and Sagi, 2013). In addition to their use as fresh vegetables, many halophytes can be transformed in profitable oilseed crops. For example, *Salicornia bigelovii*, is interesting due to its seed yield, about 2 tons per hectare per year, which are equivalent to those of conventional oilseed crops such as soybean. Seed protein and oil contents are relatively high, about 30% each, and the oil contains a high percentage of polyunsaturated fatty acids (70% of linoleic acid) so that it can be considered as high quality edible oil.

Another species to focus on to is quinoa (*Chenopodium quinoa*). This species cannot be considered as a ‘new’ crop, as it was domesticated in the Andean region between 4000 and 5000 years ago (Pickersgill, 2007). Quinoa is a ‘pseudocereal,’ producing highly nutritious seeds rich in starch and high-quality protein, including all essential amino acids, as well as a good source of dietary fiber, minerals (phosphorus, iron, magnesium, and calcium), and vitamins. The seeds are gluten-free, so that they can be consumed by celiac patients, and are considered easy to digest (Vega-Galvez et al., 2010; FAO, 2011). In addition to its exceptional nutritional quality, quinoa shows an enormous capacity to adapt to diverse environmental conditions, including extreme habitats: it is grown at sea level and in high mountains, up to almost 4,000 m. It is frost-resistant, withstanding below-zero temperatures (–4°C), but also temperatures as high as 38°C, as well as a wide range of relative humidity (40–88%). Quinoa is extremely tolerant to salinity; it is also a very water-efficient plant, remarkably resistant to drought, producing relatively good yields with low rainfall (100–200 mm). Quinoa’s capacity to adapt to such a disparate range of environmental conditions makes this species an unparalleled candidate for cultivation in different regions all over the world: in arid or semiarid land, in coastal and inland saline soils or in high mountains. At present, the only major producers are still Andean countries, especially Peru and Bolivia, but the area of cultivation is expanding to other regions. Although the market is still small, quinoa seeds and derived products are sold at relatively high prices in western countries and can be found in the ‘bio-food’ shelves of supermarkets, and in specialized shops. Considering all its proprieties, quinoa has been defined by FAO as ‘an ancient crop to contribute to world food security’ (FAO, 2011), and the UN declared 2013 as the ‘International Year of Quinoa’. Despite its great potential value, as quinoa has not been subjected to extensive modern breeding, it should be improved regarding some important traits, for example grain yield, unequal ripening or reducing the levels of (slightly) toxic saponins – although saponins are easily eliminated by washing the seeds. Since they have different medical, cosmetic and household applications, saponins could be considered as a commercially interesting by-product of quinoa cultivation.

In general, halophytes which are edible for humans can also be used to feed animals and in most cases the choice will depend on economic and marketing reasons rather than on technical and scientific ones. On other hand, saline agriculture has also the potential to provide a wide range of commercially interesting plant-derived products. Halophytic crops could also be an alternative for biodiesel production from oleaginous seeds (e.g., Abideen et al., 2012), as a source of lignocellulosic biomass for bioethanol production (e.g., Eshel et al., 2010), or for the isolation of many different metabolites with pharmacological, nutraceutical, medical, aromatic, cosmetic or other industrial or traditional household uses. These non-food applications could be economically important, but are outside the scope of this review and will not be discussed further. Therefore, development of a ‘saline agriculture’ based on the domestication of a selected number of halophytic species, represents a complementary approach to increase agricultural production in the years ahead (Rozema and Flowers, 2008; Ruan et al., 2010; Rozema et al., 2013). Once these ‘halophytic crops’ are developed and established, they could be grown in marginal saline soils – which have never been used for agriculture as our conventional crops are all salt-sensitive – as well as in abandoned crop land affected by secondary salinization. That means that commercially grown halophytes will not compete with conventional crops for fertile land and good-quality irrigation water.

## Conclusion and Perspectives

It is clear that agriculture and food production faces at the moment one of its biggest challenges in history. Feeding 9.2 × 10^9^ people is not going to be an easy task. Up to now, apart from a few exceptions, neither traditional plant breeding nor genetic engineering has delivered widely used commercial stress-tolerant varieties. Nevertheless, research lines in progress are providing promising results and we should be confident that in the coming years the combination of both approaches will allow the improvement of AST for our major crops. Moreover, the complementary approach of promoting ‘saline agriculture’ dependent on highly salt-tolerant halophytes, could be used to reclaim salinized crop land already lost for agriculture, and also naturally saline, marginal soils, using brackish, or sea water for irrigation. This will add the advantage that they will not compete with standard crops for limited resources (i.e., good-quality irrigation water and fertile crop land).

## Conflict of Interest Statement

The authors declare that the research was conducted in the absence of any commercial or financial relationships that could be construed as a potential conflict of interest.

## Footnotes

1 www.oasefoundation.eu

2 www.seawaterfoundation.org
